# Brachydactyly type A3 may be associated with shorter stature: An observation from a Chinese pediatric sample

**DOI:** 10.1371/journal.pone.0336913

**Published:** 2025-11-13

**Authors:** Hua-Hong Wu, Ya-Qin Zhang, Cheng-Dong Yu, Fang-Fang Chen, Jun-Ting Liu, Shao-Li Li, Xin-Nan Zong

**Affiliations:** 1 Department of Growth and Development, Capital Children’s Medical Center, Capital Medical University; Capital Institute of Pediatrics, Beijing, China; 2 Department of Epidemiology, Capital Children’s Medical Center, Capital Medical University; Capital Institute of Pediatrics, Beijing, China; 3 Child Health Big Data Research Center, Capital Children’s Medical Center, Capital Medical University; Capital Institute of Pediatrics, Beijing, China; CHUK: Centre Hospitalier Universitaire de Kigali, RWANDA

## Abstract

**Background:**

Brachydactyly type A3 (BDA3), a common finger deformity, demonstrates an inverse epidemiological relationship with population height, suggesting a potential link with individual stature. We aimed to investigate the distribution of BDA3 and its association with shorter stature in Chinese children.

**Methods:**

From 2022 to 2023, we conducted a cross-sectional survey in 10 randomly selected schools in Beijing with children aged 3–18. We measured height on-site, obtained left hand-wrist X-rays, calculated predicted adult height (PAH) based on height and bone age, and diagnosed BDA3 deformity based on the X-ray images. And we compared the height and PAH between the BDA3 and Non-BDA3 groups by t-test or chi-square test, examined the association of BDA3 with shorter stature and shorter PAH using binary logistic regression model.

**Results:**

A total of 5,567 children participated, with 573 diagnosed with BDA3 (a detection rate of 10.3%). Notably, girls exhibited a significantly higher detection rate than boys (14.5% vs. 6.3%). The detection rate in children <6 years was twice that in those > 12 years(15.3% vs. 7.6%). The average height and PAH were 0.30 SD and 0.22 SD lower, and the risk of shorter stature and shorter PAH were 1.57 times and 1.47 times higher in the BDA3 group than in the Non-BDA3 group, respectively. And, children >12 years in the BDA3 group had a significantly lower PAH than those in the Non-BDA3 group (about 2.0 cm). Conclusion: Children with BDA3 are more likely to have shorter stature and shorter PAH than those with no BDA3 in Chinese children aged 3–18 years.

## Introduction

Brachydactyly type A3 (BDA3) is a common finger deformity that has received limited attention in the clinical and research fields due to its minimal impact on hand function. Globally, regions with shorter heights, such as Japan, exhibit a higher detection rate of BDA3, about 25.6%, while regions with taller heights, such as Northern Europe, showed a lower rate of less than 5.0% [1,2]. These findings suggest a potential link between BDA3 and height. Our previous research reported a 27.2% detection rate of BDA3 in short-stature children and a 16.7% detection rate in shorter normal children from pediatric clinics [3]. Using large-scale, population-based data, we aimed to investigate the distribution of BDA3 and its association with shorter stature from an epidemiological perspective, offering new insights into short stature and related deformities.

## Materials and methods

We conducted a population-based cross-sectional survey in Beijing from 10/9/2022–20/5/2023, randomly selecting 10 schools with children aged 3–18 years. A questionnaire collected information on gender, age, and medical history. Height was measured on site by trained staff, and left-hand wrist radiographs were obtained using a mobile X-ray device (KBA-1, MEDNOVA, China) [4]. The studies were approved by Ethics Committee at Capital Institute of Pediatrics, Beijing. (Approval Number: SHERLL2022043).Written informed consent for participation in this study was provided by the participants’ legal guardians.

BDA3 was diagnosed when the middle phalanx of the fifth finger was shorter than half of the middle phalanx of the fourth finger [5]. Using Chinese growth references [6], children were classified by height standard deviation score (HtSDS) into shorter (HtSDS < −1) and normal (HtSDS ≥ −1) stature groups. Bone age was assessed using the TW3-C-RUS method, and predicted adult height (PAH) was calculated accordingly [7]. Based on the PAH standard deviation score (PAHSDS), children were also divided into shorter (PAHSDS < −1) and normal (PAHSDS ≥ −1) PAH groups. Genetic height height was calculated as (Father’s height (cm) + Mother’s height(cm))/2 + 6.5 cm for boys and (Father’s height (cm) + Mother’s height(cm))/2–6.5 cm for girls.

**Fig 1:**
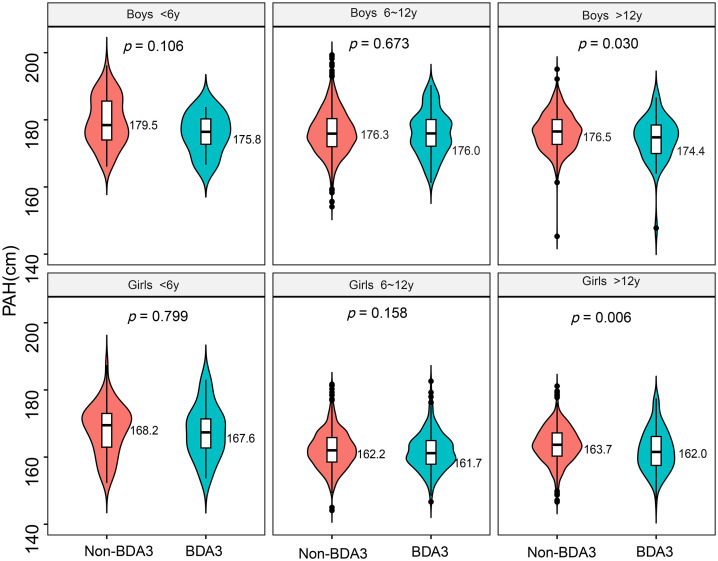
Comparison of PAH between the BDA3 and Non-BDA3 groups by gender and age. The numbers on the right side of the figure indicate the mean PAH for each group. The P-values above show the statistical significance of the PAH difference between the BDA3 and Non-BDA3 groups. Note: PAH, predicted adult height; BDA3, brachydactyly type A3.

Statistical analyses were conducted using IBM® SPSS Statistics (Version 22.0) and R software (Version 4.4.1; Posit Software). Continuous variables were tested for normality and are presented as mean ± standard deviation. Differences between the BDA3 and non-BDA3 groups were compared using independent samples t-tests. The detection rate of BDA3 across different genders and age groups is summarized as counts and percentages [N(%)], and group differences in detection rate were assessed using chi-square (χ²) tests. The association of BDA3 with shorter stature and shorter PAH were evaluated using binary logistic regression models, adjusting for gender, genetic height and bone age as covariates. Odds ratio (OR) greater than 1 indicated that BDA3 was a risk factor for shorter stature and shorter PAH. All statistical analyses described above were performed in SPSS software. Finally, comparisons of PAH among children of different genders and age groups were visualized using a violin plot, which was generated using R software (Fig 1). A two-sided p-value < 0.05 was considered statistically significant.

## Results

### The detection rate and distribution of BDA3

A total of 5,567 children aged 3–18 years (51.2% boys vs. 48.8% girls) participated, with 573 diagnosed with BDA3 (a detection rate of 10.3%). Notably, girls exhibited a significantly higher detection rate than boys (14.5% vs. 6.3%). The detection rate in children <6 years was twice that in those > 12 years (Table 1).

**Table 1:** Basic characteristics of the children in the Non-BDA3 and BDA3 groups.

	Non-BDA3 (n = 4994)	BDA3 (n = 573)	*t/χ* ^*2*^	*P*
**Gender**				
Boys	2670 (93.7)	180 (6.3)	100.40	<0.001
Girls	2322 (85.5)	393 (14.5)		
**Age (y)**	11.03 ± 3.13	10.12 ± 3.11	6.57	<0.001
< 6 y	133 (84.7)	24 (15.3)	29.04	<0.001
6–12 y	2892 (88.2)	387 (11.8)		
> 12 y	1969 (92.4)	162 (7.6)		
**Bone age (y)**	10.93 ± 3.14	9.98 ± 3.17	6.88	<0.001
**Height (cm)**	148.0 ± 17.9	140.9 ± 17.6	8.93	<0.001
**PAH (cm)**	170.2 ± 8.9	166.2 ± 8.8	10.10	<0.001
**HtSDS**	0.51 ± 1.03	0.21 ± 1.08	6.54	<0.001
**PAHSDS**	0.55 ± 1.02	0.33 ± 1.10	4.78	<0.001

Note: BDA3, brachydactyly type A3; HtSDS, height standard deviation score; PAH, predicted adult height; PAHSDS, PAH standard deviation score.

### BDA3 children have shorter stature and PAH than Non-BDA3 children

As shown in Tables 1 and 2, the BDA3 group had significantly lower mean HtSDS and PAHSDS (by 0.30 SD and 0.22 SD, respectively) and a significantly higher prevalence of both shorter stature and shorter PAH compared to the Non-BDA3 group. On comparing by gender and age in Fig 1, children >12 years in the BDA3 group had a significantly lower PAH than those in the Non-BDA3 group (about 2.0 cm).

**Table 2:** The proportion of shoter stature and shorter PAH in BDA3and Non-BDA3 group.

	Non-BDA3, n (%)	BDA3, n (%)	χ2	*P*
**Shorter stature**				
HtSDS ≥ −1	4645 (93.0)	499 (87.1)	25.711	<0.001
HtSDS < −1	349 (7.0)	74 (12.9)		
**Shorter PAH**				
PAHSDS ≥ −1	4706 (94.2)	516 (90.1)	15.455	<0.001
PAHSDS < −1	288 (5.8)	57 (9.9)		

Note: BDA3, brachydactyly type A3; HtSDS, height standard deviation score; PAHSDS, PAH standard deviation score

### BDA3 is a risk factor for shorter stature and PAH

As shown in Table 3, the BDA3 group had 1.57 times higher odds for shorter stature and 1.47 times higher odds for shorter PAH compared to the Non-BDA3 group. After adjusting for gender, genetic height, and bone age, BDA3 remained an independent risk factor for both shorter stature and shorter PAH

**Table 3:** The association of BDA3 with shorter stature and shorter PAH using logistic regression model.

	Shorter stature		Shorter PAH	
	OR (95% CI)	*p*-value	OR (95% CI)	*p*-value
**BDA3**				
Non-BDA3	1 (Reference)		1 (Reference)	
BDA3	1.57 (1.16 - 2.12)	0.003	1.47 (1.06 - 2.04)	0.020
**Gender**	0.03 (0.02–0.05)	<0.001	0.09 (0.05 - 0.14)	<0.001
**Bone age(years)**	0.79 (0.76–0.82)	<0.001	0.987(0.93 - 1.01)	0.169
**Genetic height (cm)**	0.77 (0.75 - 0.80)	<0.001	0.79 (0.76 - 0.82)	<0.001

Note:OR, Odds Ratio; CI, Confidence Interval; SDS, Standard Deviation Score. Genetic height is (Father’s height (cm) + Mother’s height(cm))/2 + 6.5 cm for boys and (Father’s height (cm) + Mother’s height(cm))/2–6.5 cm for girls.

## Discussion

We found that the proportion of BDA3 in Chinese children was 10.3%, which is higher than those in most countries in Northern Europe but lower than that in Japan. The proportion was higher in girls than in boys, consistent with the findings of Japanese studies [2]. The mechanism behind this sex difference remains unclear, but our previous research showed a surprisingly higher detection rate (42.9%) in girls with Turner syndrome [3], suggesting a potential link between BDA3 and the X chromosome, which warrants further genetic or hereditary validation. Additionally, the proportion of BDA3 tends to decrease with age, which also necessitates a longitudinal observation study. Notably, children with BDA3 are more likely to have shorter stature and PAH. For BDA3 children with PAH below the lower limit of normal, recombinant human growth hormone can be administered. We have previously confirmed that BDA3 children can still benefit from this therapy, improving their height [3].

One limitation of this study is its cross-sectional design. Longitudinal studies are needed to verify age-related changes in BDA3. Further mechanistic studies are also required to explore the genetic basis of the association between BDA3 and height.

## Conclusions

Children with BDA3 are more likely to have shorter stature and shorter PAH than those with no BDA3 in Chinese children aged 3–18 years.
